# Anticancer effects of herbal medicines in pancreatic ductal adenocarcinoma through modulation of steroid hormone response proteins

**DOI:** 10.1038/s41598-022-14174-1

**Published:** 2022-06-14

**Authors:** Zhiyi Zhang, Juan Wang, Bingying Liu, Yu Liu, Xiaowei Shi, Wenli Li, Huawei Xin, Jie Xin, Chunxiang Hao

**Affiliations:** 1 School of Pharmacy, Linyi University, Landshan District, Linyi City, 276000 Shandong Province People’s Republic of China; 2 Coastal Salinity Tolerant Grass Engineering and Technology Research Center, Ludong University, Yantai, People’s Republic of China

**Keywords:** Cancer, Computational biology and bioinformatics

## Abstract

Many individual herbs and herbal formulae have been demonstrated to provide safe and effective treatment for pancreatic ductal adenocarcinoma (PDAC); however, the therapeutic mechanisms underlying their effects have not been fully elucidated. A total of 114 herbal formulae comprising 216 single herbal medicines used to treat PDAC were identified. Cluster analysis revealed a core prescription including four herbs [*Glycyrrhizae Radix et Rhizome* (Gan Cao), *Codonopsis Radix* (Dang Shen), *Citri Reticulatae Pericarpium* (Chen Pi), and *Pinelliae Rhizoma* (Ban Xia)] in combination to treat PDAC, and 295, 256, 141, and 365 potential targets were screened for each of these four herbs, respectively. PDAC-related proteins (n = 2940) were identified from the DisGeNET database. Finally, 44 overlapping targets of herbs and PDAC were obtained, representing potential targets of the herbal medicines for PDAC treatment. GO enrichment analysis indicated that targets common to herbs and PDAC primarily functioned in response to steroid hormones. KEGG pathway enrichment analysis indicated that the herbs may prevent PDAC by influencing apoptotic, p53, and PI3K/Akt signaling pathways. Further, molecular docking analysis indicated that of identified bioactive compounds, stigmasterol, phaseol, perlolyrine, shinpterocarpin, and licopyranocoumarin have good binding ability with proteins involved in responses to steroid hormones, while stigmasterol, phaseol, perlolyrine, and DIOP have good binding ability with PTGS2(also known as COX-2), ESR1, ESR2, AR, and PGR. The anti-PDAC activity of herbal medicines may be mediated via regulation of proteins with roles in responses to steroid hormones. This study provides further evidence supporting the potential for use of herbal medicines to treat PDAC.

## Introduction

Despite decades of research, the outcomes of patients with pancreatic ductal adenocarcinoma (PDAC) remain poor. Compared with favorable prognosis tumors, PDAC is characterized by its high degree of malignancy, insidious onset, no typical symptoms, many anatomical sites, low resection rate, high recurrence rate and poor prognosis. PDAC patients have an average 5-year survival rate of less than 10%, and less than 3% for patients with advanced or metastatic diseasee. The incidence of PDAC continues to increase and there is an urgent need to improve survival rates.

Complementary and alternative medicines that are effective for PDAC patients include gene therapy, immunotherapy, targeted therapy, neoadjuvant therapy and natural medicine/herbal medicine^1^. Among complementary and alternative medicines, natural products/herbal medicines, such as Chinese herbal medicines, have become the choice of more advanced cancer patients due to their good therapeutic effects and fewer side effects^1^. For example, Ukrain(NSC-631570) is a semisynthetic compound of thiophosphoric acid and the alkaloid chelidonine derived from the plant Chelidonium majus, a common weed in Europe and western Asia. It has been shown to be effective against a range of cancers, including PDAC trials^2–4^. It is also important to mention that large amounts of polyphenols (curcumin, quercetin, green tea flavanols, resveratrol and triacetylresveratrol) have been shown to have potent antitumor, anti-inflammatory, antioxidant and pro-apoptotic effects on various human cancers, especially on PDAC models^5–7^.

In the United States, about 50–60% of cancer patients use agents from plants entirely or in combination with traditional treatment regimens such as chemotherapy and radiation therapy^8^. Gemcitabine, a nucleoside analogue, is considered to be one of the most important chemotherapeutic agents for the treatment of PDAC. However, due to the development of chemical resistance, it shows low reaction rate and disease free survival. Over the past few years, many plants and plant derivatives have been used in combination with gemcitabine, showing promising anticancer results by targeting many signaling pathways PDAC models in vitro and in vivo^9,10^. The combined use of herbal medicines with conventional chemotherapy and radiotherapy can improve the anti-cancer efficacy and reduce the side effects. Therefore, the development of new anti-PDAC drugs based on herbal medicines has a good prospect of application^11^.

Many individual herbs and herb formulae can provide safe and effective treatment for PDAC^12–14^, and recent studies support a better prognosis for patients with PDAC who receive Chinese medicine treatment compared with those undergoing conventional treatment alone^15^. Herbal formulations, including Chinese herbs, are derived from thousands of years of human experience and practical application; however, which specific herbs are beneficial for patients with PDAC requires further investigation. To release the full potential of herbal medicine for cancer therapy and broaden its application, the molecular mechanisms underlying the therapeutic activity of herbal medicine formulae in PDAC must be further explored.

The current study was designed to explore the herb combinations and underlying molecular mechanisms of traditional Chinese medicine prescriptions used to treat PDAC. In total, 114 herbal formulae tested for the treatment of PDAC in randomized controlled experiments were identified. The most frequently used herbs and the associations between use of different herbs were determined. The molecular mechanism underlying the treatment of PDAC with four core herbs frequently used in combination [*Glycyrrhizae Radix et Rhizome* (Gan Cao), *Codonopsis Radix* (Dang Shen), *Citri Reticulatae Pericarpium* (Chen Pi), and *Pinelliae Rhizoma* (Ban Xia)] was further investigated. Enrichment analysis of pathways and gene ontologies were applied to 44 protein targets common to the core herbs and PDAC. The results suggested that the four core herbs may function in PDAC treatment by influencing responses to steroid hormones, as well as apoptotic, p53, and PI3K/Akt signaling pathways. Molecular auto-dock analysis was used to validate predicted herb compound-target interactions.

## Materials and methods

### Data Sources and selection criteria

PDAC treatment-related classical prescriptions were retrieved from the published literature by screening the CNKI (https://www.cnki.net/) and PubMed (https://pubmed.ncbi.nlm.nih.gov/) databases.

Only publications meeting the following criteria were selected: randomized controlled or clinical controlled experiments to investigate the treatment of PDAC; diagnostic criteria meeting the “Guidelines for the Diagnosis and Treatment of Pancreatic Cancer in China”, formulated by the Chinese Medical Association; the treatment evaluation standard adopted was an internationally recognized universal standard; symptoms or indicators of PDAC were improved or cured following the application of traditional Chinese medicine prescriptions.

A dataset of standardized names was then compiled, by substituting all of the polysemes, synonyms, and acronyms of the herbs, according to Chinese Pharmacopoeia (2020 edition) and Chinese Materia Medica. The frequencies of occurrence of single herbs were calculated.

### Association rule and cluster analysis

Apriori algorithm-based association rule analysis was conducted and the results plotted using SPSS Modeler software. The support degree indicates the probability of simultaneous occurrence of two drugs, and the confidence degree (A → B) represents the probability of occurrence of medicine B under the condition of occurrence of medicine A. An association network diagram of 24 drugs was constructed.

Hierarchical clustering of the 24 herbs used most frequently was conducted using R.

### Establishment of a database of core herb target genes and PDAC-related genes

The Traditional Chinese Medicine Systems Pharmacology Database and Analysis Platform (TCMSP; https://tcmsp-e.com/) was searched and the chemical components and target genes of herbs retrieved.

PDAC-related genes were collected from the DisGeNET database (https://www.disgenet.org/home/) using the keywords “Pancreatic carcinoma” or “Pancreatic Ductal Adenocarcinoma”.

A protein–protein interaction (PPI) Network Map was conducted by entering targets common to retrieved herbs and PDAC into the STRING online database (https://string-db.org/); species, “*Homo sapiens*”. The resulting “tsv” file was imported into Cytoscape 3.9.1 software for further analysis of the core network.

### Gene ontology and KEGG pathway enrichment analysis

The Gene Ontology and Kyoto Encyclopedia of Genes and Genomes (KEGG) pathway enrichment analysis was performed with R^16,17^. The results are displayed in bubble charts with homemade R scripts. Network construction was generated using the network visualization software, Cytoscape (ver. 3.5.0).

### Molecular docking

Download the two-dimensional (2D) structure diagram of the compound from the PubChem database, import it into Chem3D software, draw the three-dimensional structure diagram of the compound, optimize the energy, and save the structure in mol2 format. Next, import the files into AutoDockTools-1.5.6 software, add charging information and display rotatable keys, and save them in pdbqt format. Download the protein crystal structures corresponding to the core target genes from the PDB database, import PyMOL software to remove water molecules and heteromolecules, import Auto-DockTools-1.5.6 software to add hydrogen atoms, save them in pdbqt format. These compounds were used as ligands, and the proteins were used as receptors for molecular docking analysis. Autodock vina-1.1.2 (http://vina.scripps.edu/) was used to estimate the binding ability of the molecules and targets. Results were analyzed and interpreted using PyMOL software and Discovery Studio 3.5 Client.

## Results

### High frequency herbal medicines and association rule analysis for herb combinations

**Figure 1:**
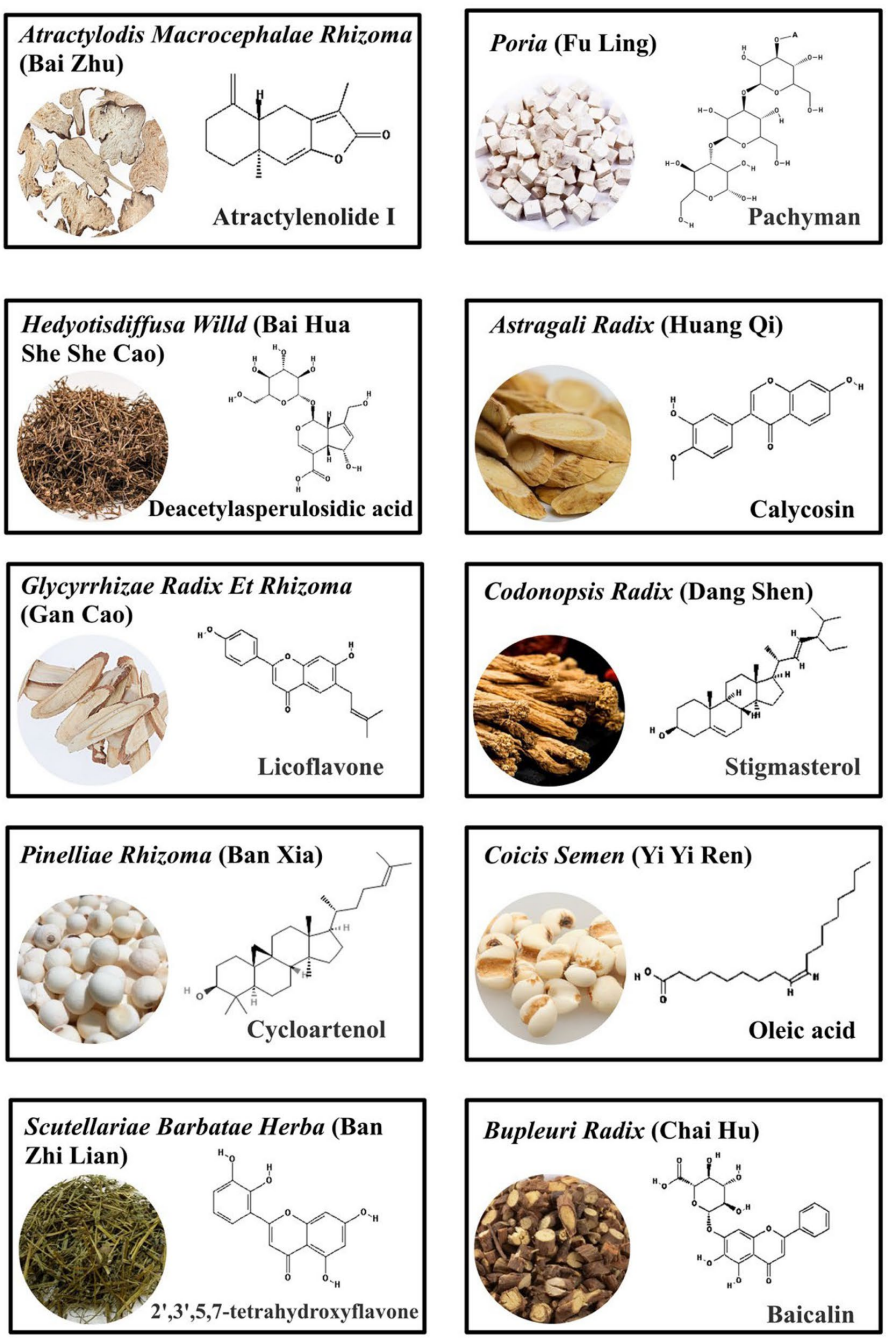
The top 10 herbs most frequently applied in treatment of PDAC and their chief chemical components with well-defined pharmacological activities. Chemical structures were downloaded from the Traditional Chinese Medicine Systems Pharmacology database and PubChem (https://pubchem.ncbi.nlm.nih.gov/). The chemical component of *Poria* (Fu Ling) shows the main chain of pachyman, including 50 $\beta$-(1 → 3) bound glucose units. “A” represent additional 47 $\beta$-(1 → 3) bound glucose units.

**Figure 2:**
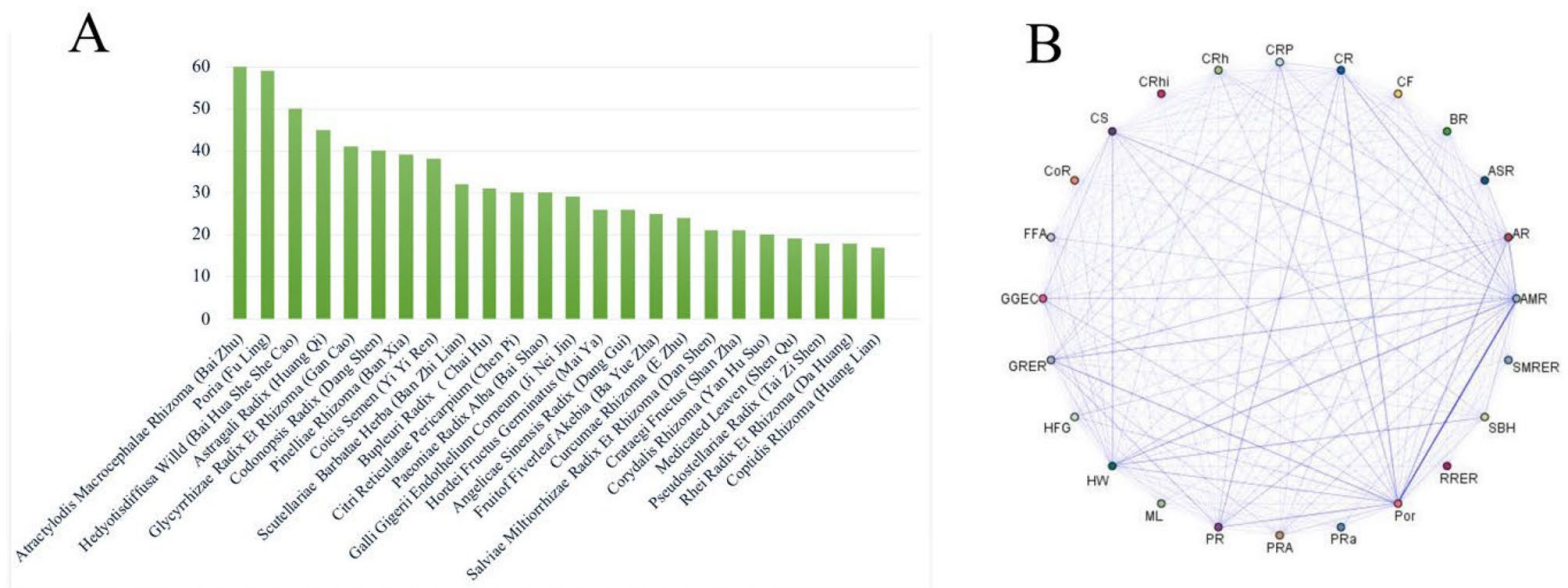
Frequently used herbal medicines and association rule analysis of herb combinations. (**A**) Frequencies of individual herbs. (**B**) Network diagram of association rules among the pharmacological mechanisms of herbs of interest.

Of the 216 herbal medicines included in the 114 prescriptions identified by literature screening, the total frequency of drug use was 1613 times (Supplementary Table 1). *Atractylodis macrocephalae Rhizoma* (Bai Zhu), *Poria* (Fu Ling), *Hedyotis diffusa Willd*. (Bai Hua She She Cao), *Astragali Radix* (Huang Qi), and *Glycyrrhizae Radix Et Rhizoma* (Gan Cao) were the herbal medicines most frequently used in the clinic (Fig. 1). Twenty-four Chinese medicines used more than 15 times were identified (Fig. 2A). These results suggest that those herbs were preferred for treatment of PDAC.

Chinese herbal medicine compatibility refers to the purposeful combination of two or more herbs, according to clinical requirements and pharmacodynamic effects, and is the main method used for clinical drug application and the basis for the composition of Chinese herbal medicine prescriptions.

Association rule analysis was carried out for 24 medicines used at high frequency using an apriori algorithm. We focused on two parameters: support and confidence level, where support was set as ≥ 20% and confidence level as ≥ 80% to obtain the top 10 herb pairs and suitable association rules (Table 1). The association between *Atractylodis macrocephalae rhizoma* (Bai Zhu) and *Poria* (Fu Ling) had the highest degree of support (52.63%), while those of *Glycyrrhizae radix et rhizom*a (Gan Cao) and *Atractylodis macrocephalae rhizoma* (Bai Zhu) with *Poria* (Fu Ling) had the highest confidence level (92%). The resulting association network diagram is presented in Fig. 2B.

**Table 1:** Apriori algorithm-based association rules for herbs used to treat PDAC.

No	Association rules	Support (%)	Confidence (%)
1	*Atractylodis macrocephalae rhizoma* (Bai Zhu) ⇒ *Poria* (Fu Ling)	52.63	81.67
2	*Poria* (Fu Ling) ⇒ *Atractylodis macrocephalae rhizoma* (Bai Zhu)	51.75	83.05
3	*Scutellariae barbatae herba* (Ban Zhi Lian) ⇒ *Hedyotisdiffusa Willd* (Bai Hua She She Cao)	28.07	81.25
4	*Citri reticulatae pericarpium* (Chen Pi) ⇒ *Poria* (Fu Ling)	26.32	80.00
5	*Astragali radix* (Huang Qi) and *atractylodis macrocephalae rhizoma* (Bai Zhu) ⇒ *Poria* (Fu Ling)	26.32	80.00
6	*Astragali radix* (Huang Qi) and *Poria* (Fu Ling) ⇒ *Atractylodis macrocephalae rhizome* (Bai Zhu)	25.44	82.76
7	*Codonopsis radix* (Dang Shen) and *atractylodis macrocephalae rhizoma* (Bai Zhu) ⇒ *Poria* (Fu Ling)	22.81	88.46
8	*Glycyrrhizae radix et rhizoma* (Gan Cao) and *atractylodis macrocephalae rhizoma* (Bai Zhu) ⇒ *Poria* (Fu Ling)	21.93	92.00
9	*Coicis semen* (Yi Yi Ren) and *atractylodis macrocephalae rhizoma* (Bai Zhu) ⇒ *Poria* (Fu Ling)	21.93	82.00
10	*Hedyotisdiffusa willd* (Bai Hua She She Cao) and *Poria* (Fu Ling) ⇒ *Atractylodis macrocephalae rhizoma* (Bai Zhu)	21.05	91.67

### Rules for combination of herbal medicines based on cluster analysis

**Figure 3:**
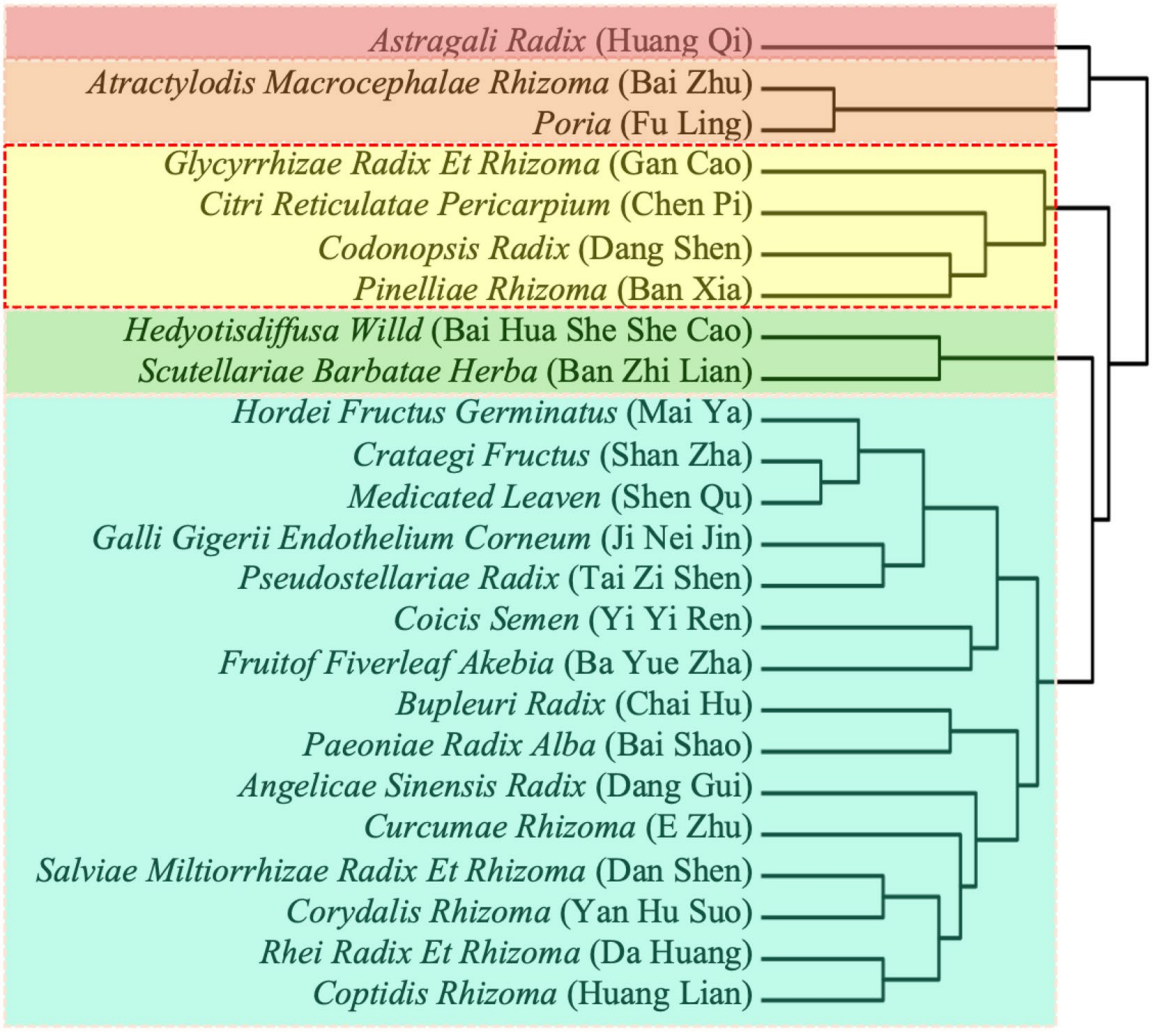
Unsupervised hierarchical cluster analysis of the 24 most frequently used herbs.

Clustering classification is widely used to determine the compatibility of herbs and the rules for combination of different Chinese medicines. Here, we applied hierarchical cluster analysis to identify the core herbs that are used in combination for treatment of PDAC. The 24 drugs mentioned in the previous section at the highest frequency were classified into five categories according to traditional Chinese medicine theory (Fig. 3). Based on compatibility rules and clinical experience, the core prescription used for PDAC treatment included four herbs: *Glycyrrhizae Radix et Rhizome* (Gan Cao), *Codonopsis Radix* (Dang Shen), *Citri Reticulatae Pericarpium* (Chen Pi), and *Pinelliae Rhizoma* (Ban Xia). Our results showed that these four herbs, which are frequently used in the clinic, are often used in combination to treat PDAC.

### Identification of potential targets of core prescription for PDAC treatment

To investigate the possible mechanism underlying the core prescriptions used to treat PDAC, the targets of the four selected herbs were obtained from the TCMSP database. In total, 295, 256, 141, and 365 potential targets were identified for *Glycyrrhizae Radix et Rhizome* (Gan Cao), *Codonopsis Radix* (Dang Shen), *Citri Reticulatae Pericarpium* (Chen Pi), and *Pinelliae Rhizoma* (Ban Xia), respectively. Importantly, 84 targets were shared by the four herbs, and these were defined as the core prescription targets. Target proteins were associated with tumors and apoptosis (e.g., TP53, TNF, BAX, BCL2, CASP3, and CASP9, among others).

**Figure 4:**
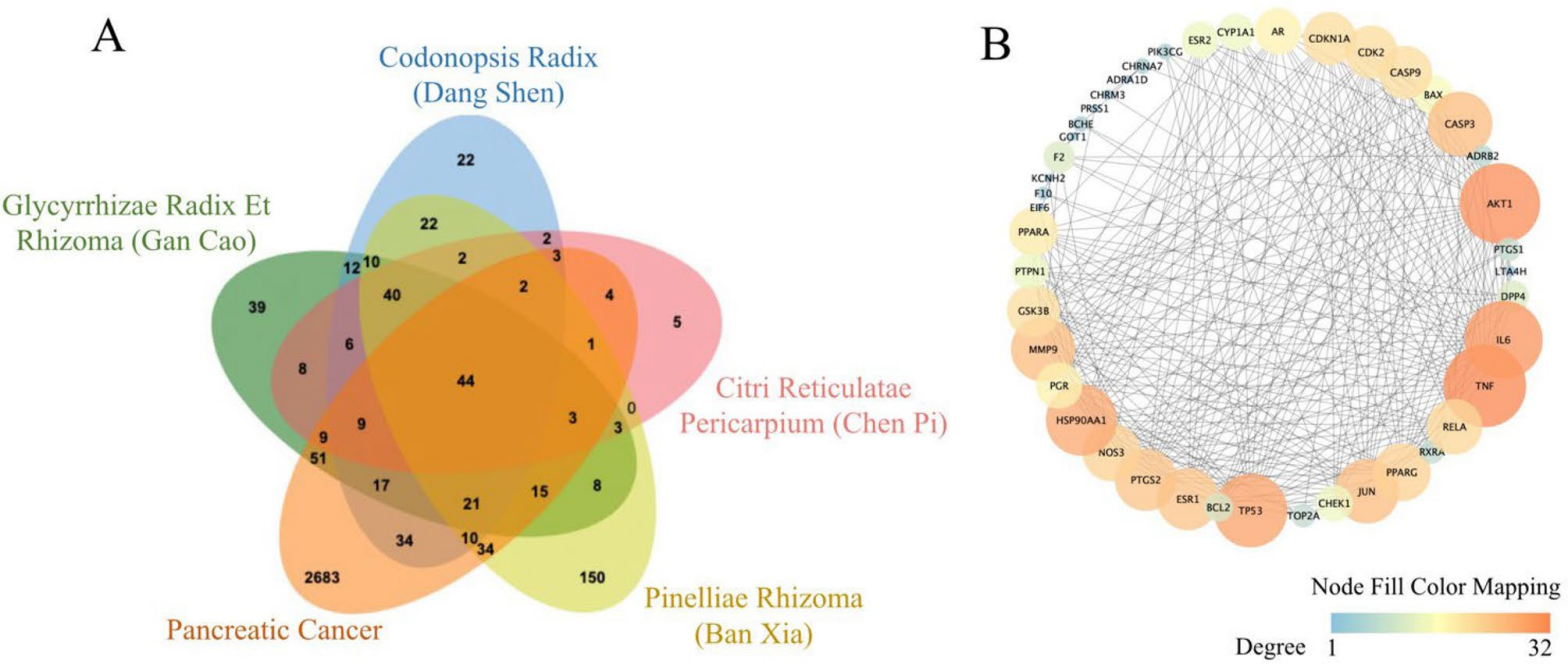
Targets of core prescriptions used for PDAC treatment. (**A**) Molecules in common between herb targets and PDAC-associated proteins. (**B**) PPI network diagram of the common targets of the four core herbs used to treat PDAC. The PPI network contains 44 nodes and 297 edges. Circles represent protein targets; orange circles indicate higher degree values. The node size of gene targets is proportional to the number of degrees.

In addition, 2940 PDAC-related proteins were identified from the DisGeNET database. Among the 84 core prescription targets, 44 overlapped with proteins in the 2940 PDAC-related group (hypergeometric *p* value < 9.04e−23; Fig. 4A). The 44 common proteins identified as both targets of the herbs and related to PDAC were considered to represent likely targets of the herbal medicines during PDAC treatment.

These 44 shared proteins were imported into STRING, and an Herb–PDAC target PPI network constructed using Cytoscape (Fig. 4B). From this PPI network, several nodes (TNF, AKT1, TP53, HSP90AA1, MMP9, JUN, CASP3, and IL6) had high degree values.

### GO and KEGG enrichment analysis

To elucidate the potential molecular mechanisms by which core prescriptions act on PDAC, GO biological process and KEGG pathway enrichment analyses were performed using the 44 identified core proteins.

**Figure 5:**
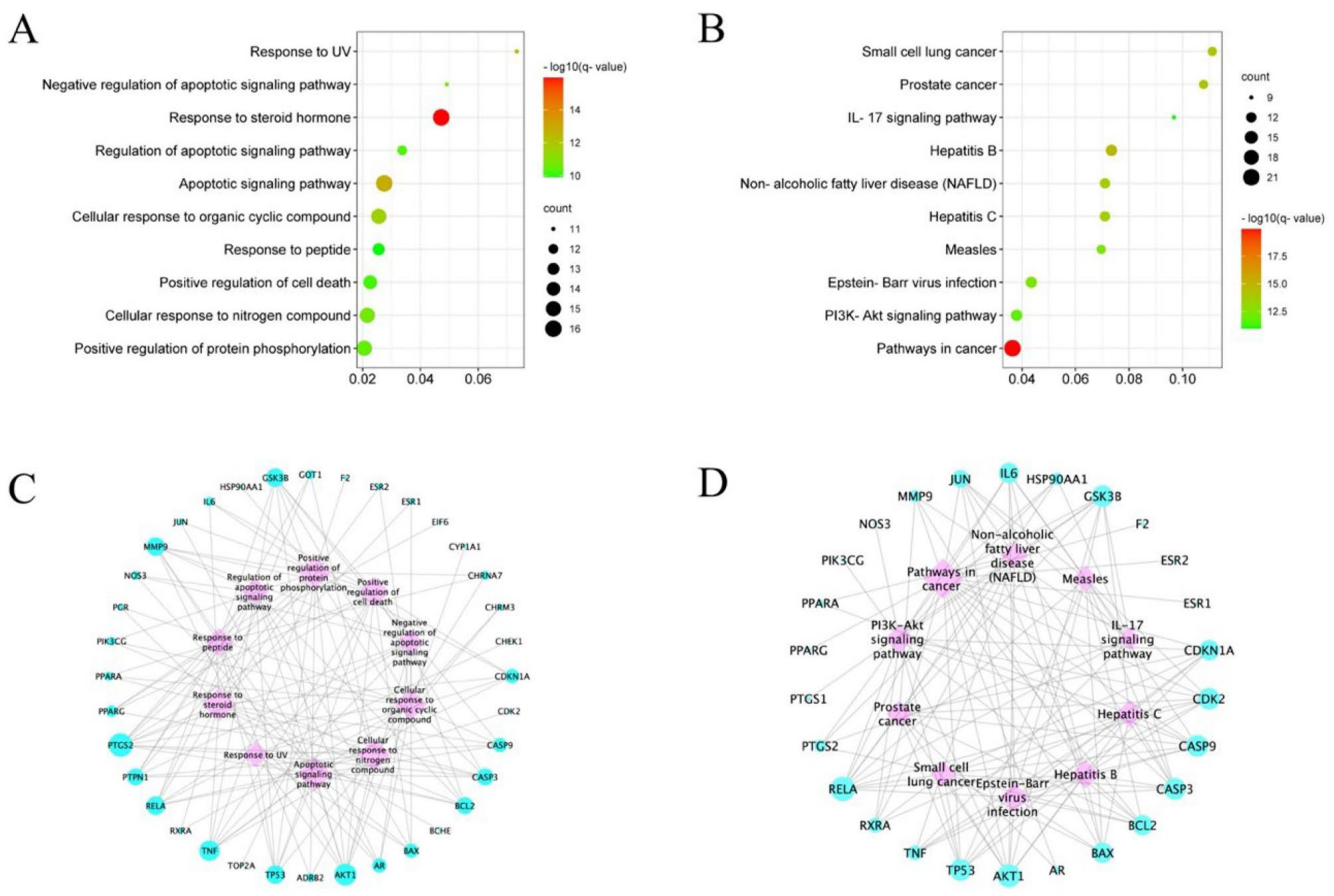
Functional analysis of common targets. (**A**) GO enrichment analysis of putative targets. (**B**) Target–GO network terms. (**C**) KEGG pathway enrichment analysis of putative targets. (**D**) Target–Signaling pathway network. Pink diamond nodes represent main signaling pathways and blue circle nodes refer to putative common targets of the four core herbs used for treatment of PDAC. Node size is proportional to the number of degrees.

The top 10 enriched GO biological process terms were determined (Fig. 5A), and analysis showed that the targets were closely related to processes involved in responses to steroid hormones and apoptotic signaling pathways. The most significantly enriched KEGG pathways included those involved in cancer, hepatitis B, apoptosis, p53 signaling, and PI3K/Akt signaling (Fig. 5B).

Target–GO term and Target–KEGG pathway networks were then constructed, based on the targets involved in each GO term or KEGG pathway. The Target–GO network comprised 46 nodes and 138 edges (Fig. 5C). The majority of targets were primarily implicated in responses to steroid hormones and apoptotic signaling pathways. In addition, the targets participating in the largest number of terms were PTGS2 (also known as COX-2), AKT1, and TNF, which were involved in 10, 9, and 8 GO terms, respectively. The Target–KEGG network included 36 nodes and 126 edges (Fig. 5D).

**Figure 6:**
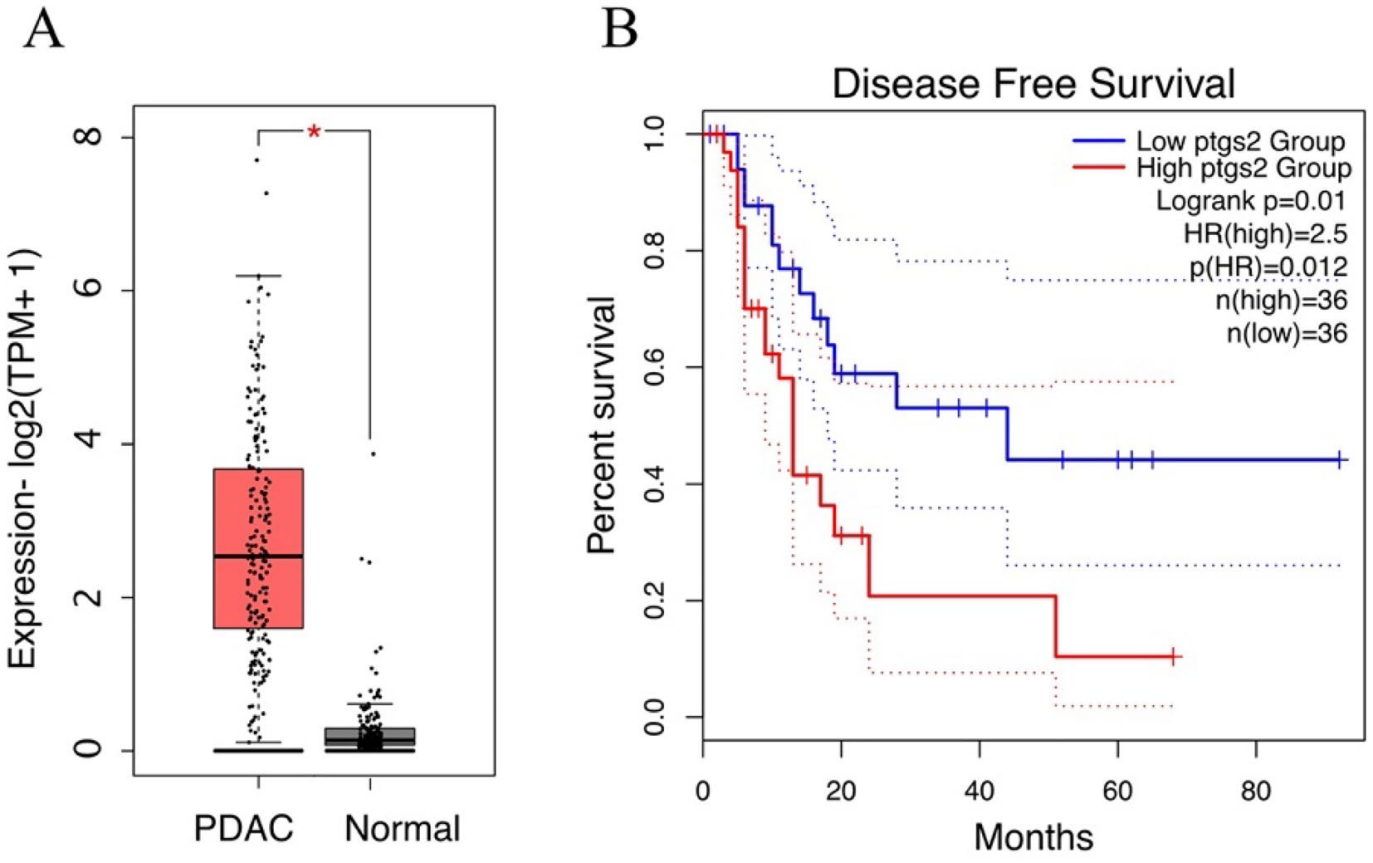
PTGS2 is highly expressed in PDAC and is associated with disease free survival. (**A**) The expression profile of PTGS2 from the TCGA Research Network (http://cancergenome.nih.gov/). Data were presented by box plots. n = 179 for PDAC tissues and n = 171 for adjacent normal tissues. TPM, transcripts per million (**B**) Kaplan–Meier survival curves comparing PDAC patients with high (80%) and low (20%) expression of PTGS2. HR, hazard ratio.

The steroid hormone response genes expression level between tumor and normal samples were extracted from GEPIA^18^. Surprisingly, mRNA expression of PTGS2 was specifically significantly upregulated in 3 types of cancer samples (among 30 types of cancer) including PDAC samples compared with normal samples (FC > 2, *p* < 0.01, Fig. 6A, Supplementary Fig. 1A). Importantly, patients with high PTGS2 expression in their tumors had poor prognosis compared to patients with low PTGS2 expression (*p* = 0.012, Fig. 6B).

The results suggest that the mechanism of action of core prescriptions for treatment of PDAC involves stimulation of responses to steroid hormones and apoptotic.

### Molecular docking

To evaluate whether active compounds from core prescription components that possess good pharmacokinetic properties could bind directly to proteins involved in responses to steroid hormone, we applied molecular docking analysis to explore potential binding modes. The top 10 compounds with highest oral bioavailability and drug-likeness values for each herb were identified as active compounds, and included flavonoids, alkaloids, amino acids, steroids, and volatile oils, among other substances (Supplementary Table 2).

**Figure 7:**
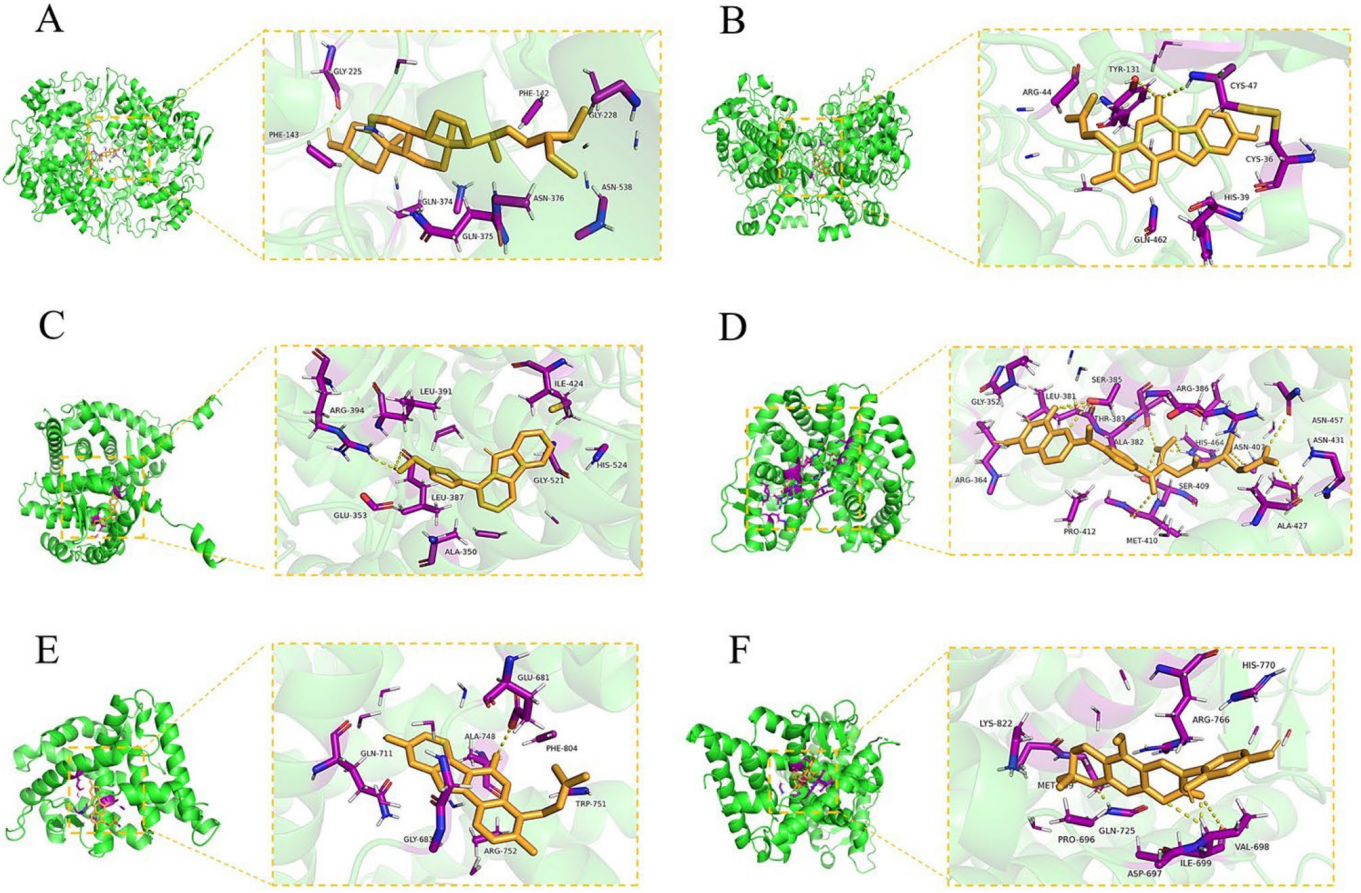
Schematic 3D representation of molecular docking models, active sites, and binding distances. Binding modes of: stigmasterol to PTGS2 (PDB id:5ikq) (**A**), phaseol to PTGS2 (**B**), perlolyrine to ESR1 (PDB id: 1a52) (**C**), DIOP to ESR2 (PDB id: 3ols) (**D**), phaseol to AR (PDB id:1e3g) (**E**), and licopyranocoumarin to PGR(PDB id: 3g8o) (**F**).

As shown in Fig. 7A, stigmasterol could bind to PTGS2 with the lowest binding energy (− 10.2 kcal/mol). The binding site of stigmasterol in PTGS2 was GLY-225. Further, the binding sites for phaseol in PTGS2 were TYR-130 and VAL-47 and the binding energy for phaseol with PTGS2 was − 10.1 kcal/mol (Fig. 7B). These results suggest that stigmasterol and phaseol could directly bind to PTGS2.

Furthermore, perlolyrine could bind to ESR1 with a binding energy of − 8.8 kcal/mol and DIOP bind to ESR2 with the same binding energy (Fig. 7C,D). Notably, phaseol and AR were able to bind with a free binding energy of − 8.6 kcal/mol, while the free binding energy of licopyranocoumarin with PGR was − 9.7 kcal/mol (Fig. 7E,F).

These results indicate that several active compounds from the four identified medicines could bind to proteins that function in responses to steroid hormones.

## Discussion

Plant-based medicines and plant-derived products remain the main source of therapeutics for much of the world’s population. Traditional Chinese medicine involves combinations of numerous co-occurring biologically active compounds, and is a valuable source of therapeutic drugs that have been used for a relatively long period of history^19^. The medicinal compatibility model can reduce the number of targets used to identify active ingredients, which makes the arbitrariness of the drug discovery process more efficient and effective^20^. Indeed, more than 60% of current anticancer chemotherapeutic drugs used in the clinic were initially developed from natural products/herbal medicines^21^.

The current study determined effective herbal prescriptions for PDAC treatment by conducting a comprehensive analysis, based on clinical cases, integration of association rules, cluster analysis, network pharmacology, bioinformatic methods, and molecular auto-dock analysis.The results indicate that *Atractylodis macrocephalae Rhizoma* (Bai Zhu), *Poria* (Fu Ling), *Hedyotis diffusa Willd.* (Bai Hua She She Cao), *Astragali Radix* (Huang Qi), and *Glycyrrhizae Radix Et Rhizoma* (Gan Cao) are the herbs most frequently used for PDAC treatment. Importantly, several recent studies have suggested that herb combinations including *Atractylodis macrocephalae Rhizoma* (Bai Zhu), *Poria* (Fu Ling), *Astragali Radix* (Huang Qi), and *Glycyrrhizae Radix Et Rhizoma* (Gan Cao) can significantly improve symptoms in patients with cancer and attenuate metastatic potential^22,23^. The effectiveness of evidence-based strategies in selecting herbs for further treatment can be determined by their efficacy. To our best knowledge, this is the first report of a potential core combination of herbs used in prescriptions for treatment of PDAC.

Data accumulated from several studies have demonstrated that the use of herbs in combination may have greater pharmacological efficacy than the use of herbs singly^24–26^. The current research reveals that combinations including four herbs used in Chinese medicine formulae, including *Glycyrrhizae Radix et Rhizome* (Gan Cao), *Codonopsis Radix* (Dang Shen), *Citri Reticulatae Pericarpium* (Chen Pi), and *Pinelliae Rhizoma* (Ban Xia), are potentially useful for the treatment of PDAC.

To further investigate the molecular mechanisms underlying the effects of combinations use of these four core herbs for treatment of PDAC, we selected 44 common targets shared by the four core herbs and PDAC, that were identified via network pharmacology analyses. GO enrichment analysis indicated that the 44 drug and disease targets in common were involved in modulation of cellular processes involved in responses to steroid hormones and apoptotic signaling pathways. Based on KEGG pathway enrichment analysis, our data further reveal that various oncogenic pathways with significant roles in the pathology of PDAC are significantly enriched for the selected common targets, including cancer, hepatitis B, apoptosis, p53 signaling, and PI3K/Akt signaling pathways.

The sex steroid hormones, estrogen, androgen, and progestin, all have functional roles in the healthy pancreas. PDAC, which is a highly lethal disease, has a higher prevalence in men than women. Together, these factors may suggest a potential role for sex hormone regulation or dysregulation in pancreatic carcinogenesis. In fact, in addition to PTGS2, the mRNA expression of AR was also significantly upregulated in PDAC samples (and other five cancers) compared with normal samples (FC > 1.6, *p* < 0.01, Supplementary Fig. 1B).

The results of molecular docking showed that stigmasterol and phaseol had high affinity for PTGS2. PTGS2 is an inducible enzyme with vital roles in the pathophysiological processes of PDAC^27^. Importantly, there is evidence that PTGS2 is an attractive target in PDAC because it is highly upregulated and participates in anti-apoptotic mechanisms^28^. Our data indicate that perlolyrine can bind to ESR1 and DIOP to ESR2 with binding energies of − 8.8 kcal/mol. A recently published study revealed that the G protein-coupled estrogen receptor (GPER) inhibits PDAC^29^. It was demonstrated that GPER acts as a tumor suppressor in cancers that are not classically considered hormone responsive, suggesting that GPER activity may contribute to biological differences between the sexes that influence cancer progression and response to modern therapies^29^. An additional study showed that there is an association between thyroid hormone supplementation and PDAC invasion^30^.

In summary, the results of the current study suggest that four core herbs may play important roles in treating PDAC by regulating proteins that function in response to steroid hormones. Hence, bioactive compounds, such as stigmasterol, phaseol, perlolyrine, and DIOP, may exert anti-cancer effects against PDAC.

## Conclusion

This study reveals the core prescription used for PDAC treatment included four herbs: *Glycyrrhizae Radix et Rhizome* (Gan Cao), *Codonopsis Radix* (Dang Shen), *Citri Reticulatae Pericarpium* (Chen Pi), and *Pinelliae Rhizoma* (Ban Xia). The anti-PDAC activity of core prescription may be mediated via regulation of proteins with roles in responses to steroid hormones. This study provides further evidence supporting the potential for use of herbal medicines to treat PDAC.

## Supplementary Information

Supplementary Table 1.

Supplementary Table 2.

Supplementary Figure 1.

## Data Availability

The original contributions presented in the study are included in the article/Supplementary Material, further inquiries can be directed to the corresponding authors.

## Abbreviations

AMR: *Atractylodis Macrocephalae Rhizoma* (Bai Zhu)
AR: *Astragali Radix* (Huang Qi)
ASR: *Angelicae Sinensis Radix* (Dang Gui)
BR: *Bupleuri Radix* (Chai Hu)
CF: *Crataegi Fructus* (Shan Zha)
CoR: *Corydalis Rhizoma* (Yan Hu Suo)
CRh: *Curcumae Rhizoma* (E Zhu)
CRhi: *Coptidis Rhizoma* (Huang Lian)
CR: *Codonopsis Radix* (Dang Shen)
CRP: *Citri Reticulatae Pericarpium* (Chen Pi)
CS: *Coicis Semen* (Yi Yi Ren)
FFA: *Fruitof Fiverleaf Akebia* (Ba Yue Zha)
GGEC: *Galli Gigerii Endothelium Corneum* (Ji Nei Jin)
GO: Gene ontology
GRER: *Glycyrrhizae Radix Et Rhizoma* (Gan Cao)
HFG: *Hordei Fructus Germinatus* (Mai Ya)
HW: *Hedyotis diffusa Willd* (Bai Hua She She Cao)
KEGG: Kyoto encyclopedia of genes and genomes
ML: *Medicated Leaven* (Shen Qu)
PDAC: Pancreatic ductal adenocarcinoma
Por: *Poria* (Fu Ling)
PR: *Pinelliae Rhizoma* (Ban Xia)
PRa: *Pseudostellariae Radix* (Tai Zi Shen)
PRA: *Paeoniae Radix Alba* (Bai Shao)
RRER: *Rhei Radix Et Rhizoma* (Da Huang)
SBH: *Scutellariae Barbatae Herba* (Ban Zhi Lian)
SMRER: *Salviae Miltiorrhizae Radix Et Rhizoma* (Dan Shen)
