# Supplementary material for: Anticancer effects of herbal medicines in pancreatic ductal adenocarcinoma through modulation of steroid hormone response proteins
Source: Sci Rep. 2022 Jun 14;12:9910. doi: 10.1038/s41598-022-14174-1 (PMC9198029; doi:10.1038/s41598-022-14174-1)
Supplement: Supplementary file 3 — Supplementary Figure 1. [file 41598_2022_14174_MOESM3_ESM.pdf]

A

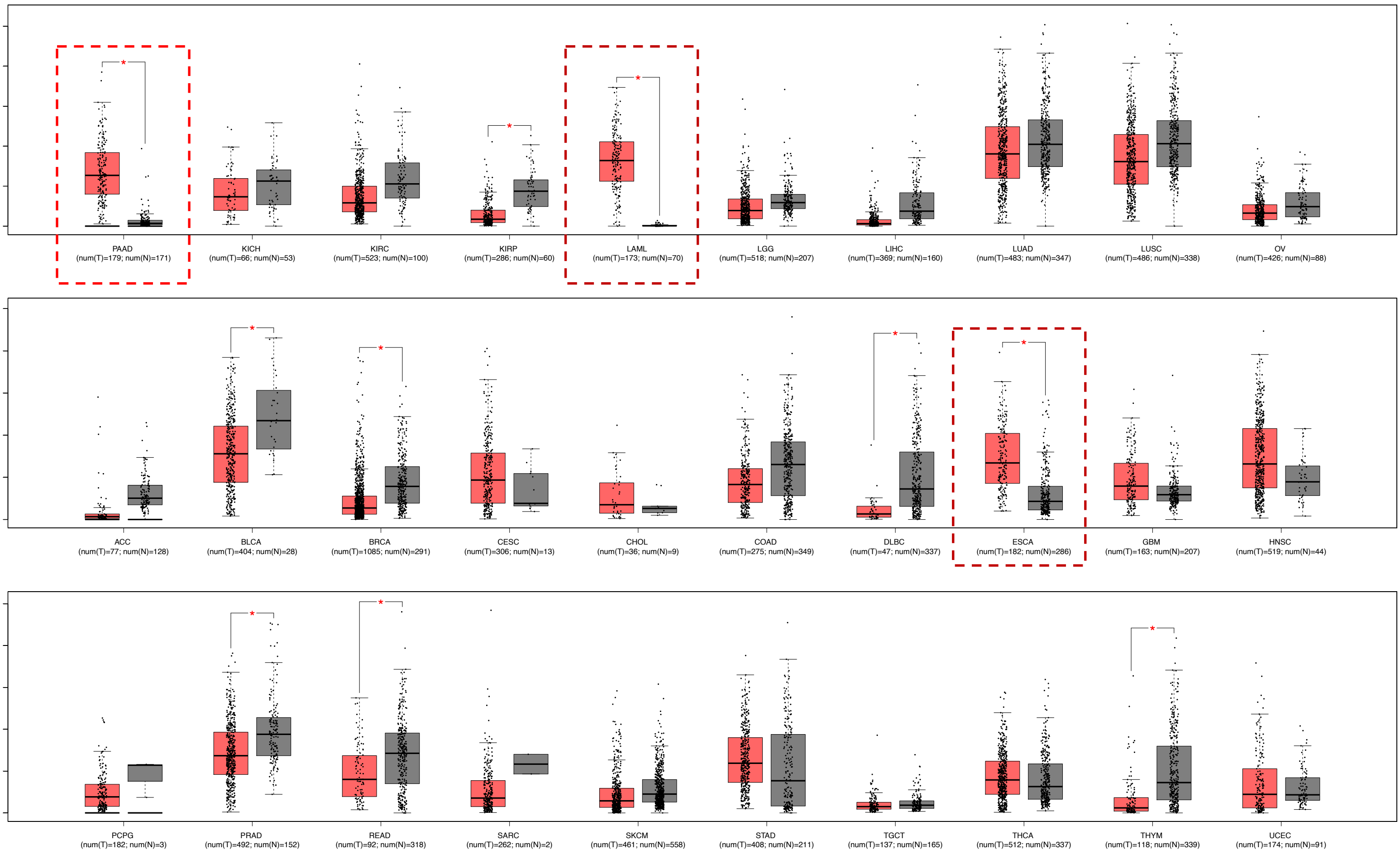
PAAD (num(T)=179; num(N)=171)
KICH (num(T)=66; num(N)=53)
KIRC (num(T)=523; num(N)=100)
KIRP (num(T)=286; num(N)=60)
LAML (num(T)=173; num(N)=70)
LGG (num(T)=518; num(N)=207)
LIHC (num(T)=369; num(N)=160)
LUAD (num(T)=483; num(N)=347)
LUSC (num(T)=486; num(N)=338)
OV (num(T)=426; num(N)=88)
ACC (num(T)=77; num(N)=128)
BLCA (num(T)=404; num(N)=28)
BRCA (num(T)=1085; num(N)=291)
CESC (num(T)=306; num(N)=13)
CHOL (num(T)=36; num(N)=9)
COAD (num(T)=275; num(N)=349)
DLBC (num(T)=47; num(N)=337)
ESCA (num(T)=182; num(N)=286)
GBM (num(T)=163; num(N)=207)
HNSC (num(T)=519; num(N)=44)
PCPG (num(T)=182; num(N)=3)
PRAD (num(T)=492; num(N)=152)
READ (num(T)=92; num(N)=318)
SARC (num(T)=262; num(N)=2)
SKCM (num(T)=461; num(N)=558)
STAD (num(T)=408; num(N)=211)
TGCT (num(T)=137; num(N)=165)
THCA (num(T)=512; num(N)=337)
THYM (num(T)=118; num(N)=339)
UCEC (num(T)=174; num(N)=91)

B

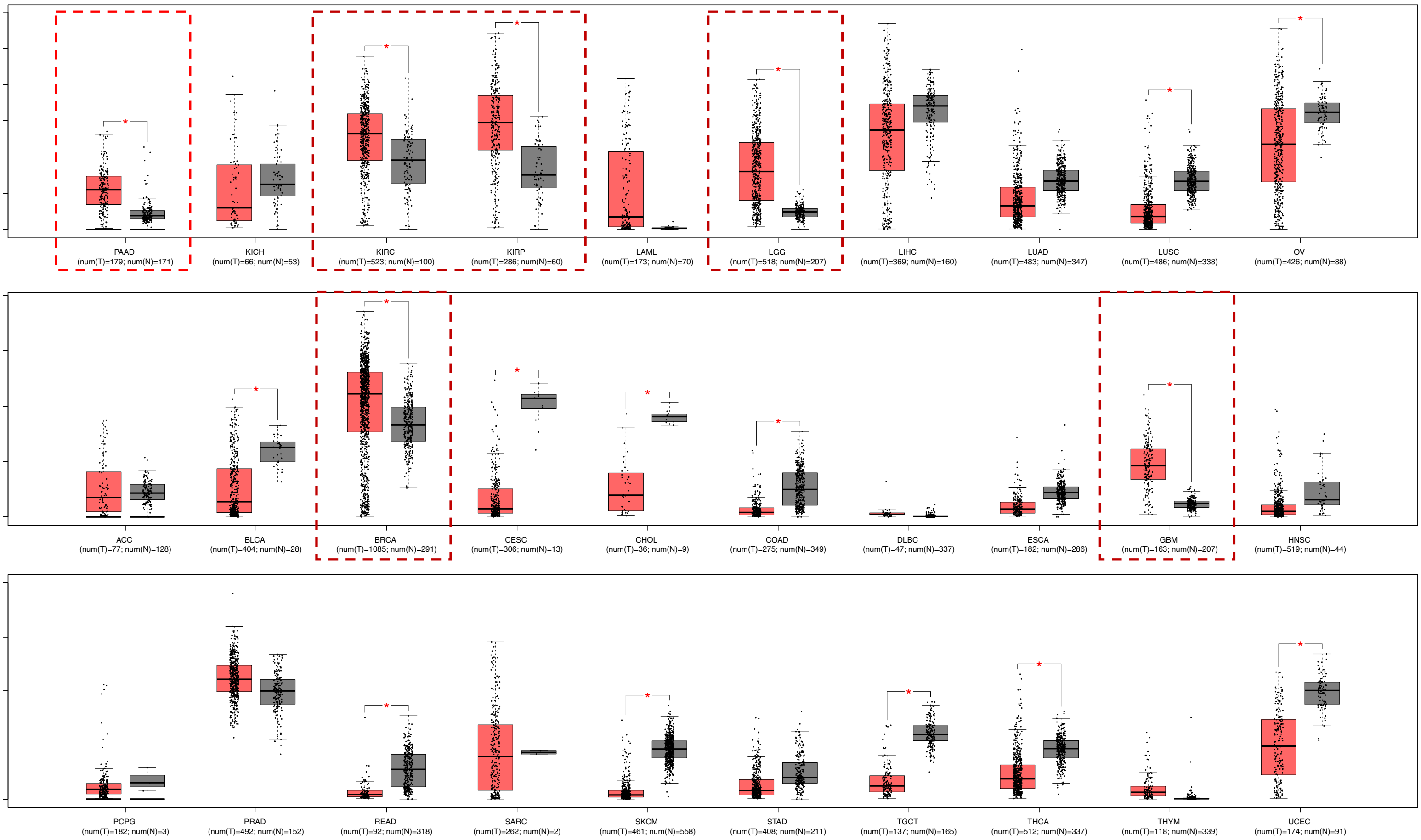
PAAD (num(T)=179; num(N)=171)
KICH (num(T)=66; num(N)=53)
KIRC (num(T)=523; num(N)=100)
KIRP (num(T)=286; num(N)=60)
LAML (num(T)=173; num(N)=70)
LGG (num(T)=518; num(N)=207)
LIHC (num(T)=369; num(N)=160)
LUAD (num(T)=483; num(N)=347)
LUSC (num(T)=486; num(N)=338)
OV (num(T)=426; num(N)=88)
ACC (num(T)=77; num(N)=128)
BLCA (num(T)=404; num(N)=28)
BRCA (num(T)=1085; num(N)=291)
CESC (num(T)=306; num(N)=13)
CHOL (num(T)=36; num(N)=9)
COAD (num(T)=275; num(N)=349)
DLBC (num(T)=47; num(N)=337)
ESCA (num(T)=182; num(N)=286)
GBM (num(T)=163; num(N)=207)
HNSC (num(T)=519; num(N)=44)
PCPG (num(T)=182; num(N)=3)
PRAD (num(T)=492; num(N)=152)
READ (num(T)=92; num(N)=318)
SARC (num(T)=262; num(N)=2)
SKCM (num(T)=461; num(N)=558)
STAD (num(T)=408; num(N)=211)
TGCT (num(T)=137; num(N)=165)
THCA (num(T)=512; num(N)=337)
THYM (num(T)=118; num(N)=339)
UCEC (num(T)=174; num(N)=91)

Supplementary Figure 1. The mRNA expression between tumor (TCGA) and normal (TCGA plus GTEX) samples across cancers. (A) The mRNA expression of PTGS2 across cancers. Three types of cancer samples in the box show significantly upregulated expression level (FC>2, p<0.01). (B) The mRNA expression of AR across cancers. Six types of cancer samples in the box show significantly upregulated expression level (FC>1.6, p<0.01).

Tumor abbreviations.

ACC	Adrenocortical carcinoma
BLCA	Bladder Urothelial Carcinoma
BRCA	Breast invasive carcinoma
CESC	Cervical squamous cell carcinoma and endocervical adenocarcinoma
CHOL	Cholangio carcinoma
COAD	Colon adenocarcinoma
DLBC	Lymphoid Neoplasm Diffuse Large B-cell Lymphoma
ESCA	Esophageal carcinoma
GBM	Glioblastoma multiforme
HNSC	Head and Neck squamous cell carcinoma
KICH	Kidney Chromophobe
KIRC	Kidney renal clear cell carcinoma
KIRP	Kidney renal papillary cell carcinoma
LAML	Acute Myeloid Leukemia
LGG	Brain Lower Grade Glioma
LIHC	Liver hepatocellular carcinoma
LUAD	Lung adenocarcinoma
LUSC	Lung squamous cell carcinoma
MESO	Mesothelioma
OV	Ovarian serous cystadenocarcinoma
PAAD	Pancreatic adenocarcinoma
PCPG	Pheochromocytoma and Paraganglioma
PRAD	Prostate adenocarcinoma
READ	Rectum adenocarcinoma
SARC	Sarcoma
SKCM	Skin Cutaneous Melanoma
STAD	Stomach adenocarcinoma
TGCT	Testicular Germ Cell Tumors
THCA	Thyroid carcinoma
THYM	Thymoma
UCEC	Uterine Corpus Endometrial Carcinoma
UCS	Uterine Carcinosarcoma
UVM	Uveal Melanoma
